# Assessing neurocognitive functioning among adults ageing with and without HIV at the Kenyan Coast: measurement issues and correlates

**DOI:** 10.3389/fnagi.2025.1702869

**Published:** 2025-12-04

**Authors:** Patrick N. Mwangala, Carophine Nasambu, Ryan G. Wagner, Mihaela Duta, Gaia Scerif, Charles R. Newton, Amina Abubakar

**Affiliations:** 1 Institute for Human Development, Aga Khan University, Nairobi, Kenya; 2 Centre for Geographic Medicine Research Coast, Kenya Medical Research Institute (KEMRI), Kilifi, Kenya; 3 School of Public Health, University of the Witwatersrand, Parktown, South Africa; 4 MRC/Wits Rural Public Health and Health Transitions Research Unit (Agincourt), School of Public Health, Faculty of Health Sciences, University of the Witwatersrand, Johannesburg, South Africa; 5 Oxford Research Software Engineering Group, Doctoral Training Centre, University of Oxford, Oxford, United Kingdom; 6 Department of Public Health, Pwani University, Kilifi, Kenya; 7 Department of Psychiatry, University of Oxford, Warneford Hospital, Oxford, United Kingdom

**Keywords:** older adults, HIV, cognition, Kenya, OCSPlus, determinants

## Abstract

**Background:**

Cognitive impairment is one of the most prevalent complications of HIV infection, with significant medical and functional impacts. However, valid, and reliable assessment tools are lacking for the newly emergent ageing population of people living with HIV (PLWH) in many parts of sub-Saharan Africa (SSA), including Kenya. Without these tools, critical intervention opportunities, e.g., psychoeducation, treatment and additional support, are missed. To bridge this gap in Kenya, we adapted the Oxford Cognitive Screen Plus (OCSPlus), a tablet-based cognitive assessment tool designed for low-literacy settings, with adults ageing with HIV ≥ 50 years and their uninfected peers. This study examines the acceptability, reliability, and validity of the OCSPlus tool among older Kenyan adults and provides an initial understanding of the cognitive performance of these adults (by HIV status) and the biopsychosocial factors associated with their cognitive performance.

**Methods:**

In a cross-sectional sample of 440 older adults (257 living with HIV), we administered the OCSPlus tool alongside the Raven’s Standard Progressive Matrices (RSPM), the International HIV Dementia Scale (IHDS), and health and sociodemographic assessments.

**Results:**

There was a high level of acceptability of OCSPlus by participants and test administrators. OCSPlus demonstrated good test–retest reliability. Acceptable correlations between individual OCSPlus sub-tasks and conventional tests (RSPM and IHDS) were also observed for convergent validity. Regarding cognitive performance, older adults living with HIV (OALWH) presented with significantly lower scores on language (picture naming task), executive function, and the IHDS overall score compared to their uninfected peers. However, OALWH performed significantly better on memory domain (orientation, word encoding and word recall tasks), non-verbal intelligence and processing speed. There were no differences in attention domain. Cognitive performance as assessed by OCSPlus was significantly associated with behavioural and lifestyle factors (physical activity, sleeping difficulties, obesity, and sexual activity), sociodemographic factors (age, sex, educational status, household income, household size and asset index), medical or treatment factors (self-reported urinary incontinence, hearing problems, history of TB, seeking services of traditional healers, antiretroviral therapy (ART) regimen change, being on 3rd line ART treatment) and psychosocial factors including ageism and food insecurity.

**Conclusion:**

We demonstrated the feasibility of OCSPlus administration by trained lay persons, its acceptability, and preliminary reliability and validity among low-literacy older adults on the Kenyan coast. Mean cognitive scores were mixed across the two groups. Cognitive performance was associated with several biopsychosocial factors spanning behavioural/lifestyle, sociodemographic, psychosocial, medical and treatment factors. Further validation studies and epidemiological research are needed to understand better the utility of OCSPlus and the cognitive function of these adults.

## Introduction

Cognitive impairment is one of the most frequently witnessed complications of Human Immunodeficiency virus (HIV) infection, affecting about 50% of people living with HIV (PLWH) globally and has important medical, functional, and public health consequences (Zenebe et al., 2022). Besides, the number of PLWH getting to 60 years old is rapidly increasing, an age at which dementia prevalence begins to rise in the general population. Given that age is an important risk factor for dementia (Livingston et al., 2020), even small effects of premature, accelerated, or accentuated neurocognitive ageing in PLWH may have serious public health consequences for dementia risk in the global HIV population. This underscores the crucial need for timely screening and intervention, which can slow cognitive decline and prevent the progression to more severe stages. Among existing studies, evidence for premature cognitive ageing is inconsistent whereas evidence for accelerated cognitive ageing appears consistent but is based on limited number of studies (Aung et al., 2021). Hence, stronger evidence is urgently needed to inform healthcare providers and the HIV sector about cognitive impairment risk among PLWH to properly facilitate screening, prevention and treatment among adults ageing with HIV.

Estimates of the prevalence for cognitive impairment among Africa’s 25.6 million PLWH (UNAIDS, 2022) range from 14 to 88% depending on geographical location, study design, measurement tools and participants’ antiretroviral therapy (ART) status (Mekuriaw et al., 2023). Cognitive deficits in HIV, known as HIV-associated neurocognitive disorder (HAND), typically cause impairments in various cognitive domains, including learning, memory, executive function, language, attention, processing speed and motor functions (Alford and Vera, 2018). HAND ranges in severity from asymptomatic and mild forms to severe dementia-type forms (Alford and Vera, 2018). There are significant medical and functional impacts associated with having even mild cognitive impairments, such as a higher risk of mortality, increased likelihood of developing a more severe impairment, limitations in activities of daily living, decreased quality of life, poor decision making and increased HIV transmission risk behaviors, placing many PLWH at risk for worse health outcomes (Gorman et al., 2009; Vivithanaporn et al., 2010; Tozzi et al., 2003; Ettenhofer et al., 2009; Iudicello et al., 2013; Thames et al., 2012). Recent findings from several settings suggest that the cognitive profile of PLWH has evolved. The advent of combination ART (cART) has lessened the severity but not the frequency of these impairments (Alford and Vera, 2018; Michael et al., 2021; Clifford and Ances, 2013). Noticeably, there has been a sharp drop in the severe forms of HAND and an increase in milder forms (Alford and Vera, 2018). Besides, the current manifestations of HAND show a subtle subcortical involvement and more cortical involvement compared to the progressive subcortical dementia with marked deterioration of cognitive speed and motor functions observed in the pre-cART era (Guha et al., 2016). Some findings also show a change in the cognitive domains most affected, from largely motor skills, processing speed, and verbal fluency in the pre-cART era to predominantly memory, learning and executive functioning in the cART era, suggesting possible domain-specific effects of HIV or cART (Heaton et al., 2011). Hence, understanding the domain-specific effects of HIV on cognition may extend our understanding of the mechanism of HAND, potentially guiding care, and future research.

As life expectancy of PLWH approaches that of people without HIV, PLWH become more vulnerable to age-associated cognitive impairment (Clifford and Ances, 2013). A combination of blood brain barrier dysfunction, legacy effect of CNS impairment prior to ART initiation, with the direct effects of HIV on the CNS, chronic neuroinflammation, telomere shortening, neurogenesis impairments and neurotoxicity associated with ART, alters and amplifies the mechanisms of normal brain aging in HIV, leading to cognitive decline and structural brain changes at an earlier age than normally witnessed in the general population (Lazar et al., 2024; Scutari et al., 2017; Irollo et al., 2021). Additionally, given the rapid increase in chronic age-related conditions in many parts of Africa, which also coincides with increasing prevalence of HIV among older adults, it is imperative to understand the cognitive profile of this vulnerable population to guide the design and implementation of health programs and policies in this population. In many countries in sub-Saharan Africa (SSA), older adults living with HIV often begin ART with severe levels of immunosuppression (Asiimwe et al., 2015). Such clinical, disease-specific or biological characteristics, along with a wide range of sociocultural factors – such as available support systems to provide care and assistance to older people - could result in different distributions of cognitive impairments in SSA compared to other regions. This underscores the need for country-specific data on cognition to facilitate adequate screening, diagnosis and treatment of these impairments.

Screening for cognitive impairment is critical to good holistic care and treatment strategies of HIV (Antinori et al., 2013). Routine screening can assist healthcare providers in detecting HAND early, determining when to adjust cART regimens, monitor cognitive function, and educate clients in a timely fashion about the effects of cognitive impairment and ways to minimize it (Antinori et al., 2013; Cysique et al., 2012; Shah et al., 2016). Nonetheless, the screening is rarely done in SSA due to numerous challenges, including the scarcity of locally developed tests and a lack of expert personnel to administer them (Mwangala et al., 2018; Gouse et al., 2021), thus missing critical opportunities to identify HAND and intervene for the millions of PLWH in the region. Additionally, many of the conventional screening measures, e.g., International HIV Dementia Scale (IHDS) take a binary view of cognition, relying on global scores with a single cut-off value for impairment. They are also subject to ceiling effects (i.e limited ability to differentiate test performance among high-scoring individuals partly due to task simplicity), low sensitivity and are inappropriate for populations with low literacy and low numeracy (Folstein et al., 1975; Hoops et al., 2009; Nasreddine et al., 2005).

In the current study, we use the Oxford Cognitive Screen Plus (OCSPlus) (Demeyere et al., 2021) and two other conventional tests the International HIV Dementia Scale – IHDS, and the Raven’s Standard Progressive Matrices (RSPM) to assess the cognitive function of older adults. The OCSPlus is a tablet-based cognitive screening instrument which bridges the gap between brief screens and comprehensive neuropsychological batteries, in terms of resource-efficiency and good psychometric properties (Demeyere et al., 2021). Unlike many conventional tests that rely on reading and numeracy skills, the OCSPlus primarily relies on visual abilities, making literacy and numeracy less critical to complete the test (Demeyere et al., 2021; Humphreys et al., 2017). The OCSPlus has previously been validated for detection of subtle neurocognitive deficits in a healthy aging population in the UK and Germany (Demeyere et al., 2021), sub-acute and chronic stroke survivors in the UK (Webb et al., 2022), patients with amnestic mild cognitive impairment in Germany (Hübner et al., 2020) and in a population of older adults in a low literacy and socioeconomic setting in South Africa (Humphreys et al., 2017). Overall, the findings from these studies have demonstrated that OCSPlus has high task compliance and good validity (construct, convergent, divergent and external validity), thus improving the measurement of cognition with minimal language content and avoiding floor and ceiling effects present in other brief cognitive assessment measures. However, some of the earlier studies have reported low convergent validity, and many of them have not examined the reliability of this tool, underscoring the need for further adaptation and validation in different settings and populations. This study focuses on Kenya, a country experiencing simultaneous demographic and epidemiologic transitions (Kenya National Bureau of Statistics, 2019). To our knowledge, no study in the country has examined the cognitive function of older adults living with HIV. To bridge this gap, the present study seeks to examines the acceptability, reliability (internal consistency and test–retest), and convergent validity of the OCSPlus tool among older Kenyan adults and provides an initial understanding of the cognitive performance of these adults (by HIV status) and the biopsychosocial factors associated with their cognitive performance.

## Materials and methods

### Study design and setting

This work is part of a larger cross-sectional study, the HIV-Associated Neurocognitive Disorders (HAND) study, which examines various neuropsychological outcomes, such as cognition, mental health, and psychosocial functioning in adults aged 50 years and older on the Kenyan coast in Mombasa and Kilifi counties (Mwangala et al., 2023a; Mwangala et al., 2023b; Mwangala et al., 2022; Mwangala et al., 2024). Data collection for this study took place between 2020 and 2021. Kilifi County has approximately 1.5 million residents, most of whom are rural dwellers and belong to the Mijikenda ethnic group (Kenya National Bureau of Statistics, 2019). Recent data indicates that Kilifi has an adult HIV prevalence of 4.5% (National AIDS Control Council (NACC), 2016). Mombasa County borders Kilifi to the north and has an estimated population of 1.2 million people (Kenya National Bureau of Statistics, 2019) and an adult HIV prevalence of 7.5% (National AIDS Control Council (NACC), 2016). More details have been described elsewhere (Mwangala et al., 2022).

### Participants recruitment

#### Older adults living with HIV

Older adults living with HIV (OALWH) were recruited from two public HIV-specialized clinics: the Comprehensive Care and Research Clinic (at the Kilifi County Hospital) and the Comprehensive Care Clinic at the Coast General Teaching and Referral Hospital in Mombasa. The two clinics were chosen because of their wide client catchment area and a large volume of potential respondents. To participate in the study, individuals had to be at least 50 years old, have a confirmed HIV seropositivity status, be on routine HIV treatment, and be willing and able to provide informed consent for their participation. Exclusion criteria included: (a) having any acute medical/psychiatric condition on the assessment day, and (b) signs and symptoms of severe physical and mental disorders such as psychotic disorders. A rapid medical assessment was completed by a clinician to ascertain this. Excluded participants were referred to access appropriate health services through the county and sub-county referral pathway.

Two community health volunteers helped us review existing records at the HIV clinics to identify potential participants. We attempted to contact all potential respondents with contact details to invite them to participate in our project. Subsequently, a research assistant introduced the study to the potential participants before enrolment. We started the participant recruitment in Mombasa County; however, it was interrupted by the COVID-19 pandemic after having recruited and assessed only 72 OALWH. The remaining participants (*n =* 368) were recruited in Kilifi County upon resumption of project activities.

#### Older adults without HIV

We used the Kilifi Health and Demographic Surveillance System (KHDSS) to identify families with eligible older adults. Potential participants were randomly identified from the existing database and approached at their homesteads using Global Positioning System (GPS) coordinates by a trained research assistant. Study information was shared with all persons who expressed interest in participating. To be included in the study, participants had to be aged at least 50 years, be resident of Kilifi County, and be willing and able to provide written informed consent, including willingness to be tested for HIV using a rapid HIV testing kit (OraQuick) for a confirmation of their HIV seronegative status.

### Sample size calculations

We estimated our sample size using a previous study (Bloch et al., 2016) that reported significant cognitive function differences between adults living with HIV and their uninfected peers. In this, we carried out power analyses in Stata (using effect estimates and proportions from previous literature). An overall sample of 218 was sufficient to identify differences at 85% power and a 5% level of statistical significance. A sample of 400 participants was deemed adequate to conduct multiple regression analyses to explore correlates of cognitive scores among these adults (Jenkins and Quintana-Ascencio, 2020). The sample size for psychometric analysis is based on recommended numbers of at least 100 participants among controls for stable estimates of reliability and internal consistency (Yurdugül, 2008).

### Measures

Our research instruments were programmed on Android tablets using the Electronic Data Capture (REDCap) platform (Harris et al., 2009) for face-to-face interviewer administration. The first author (PNM) trained the research assistants for 2 weeks. The intensive training emphasized how to administer and interpret the cognitive tests before field implementation. The training comprised various components, including familiarization with study procedures, infection prevention and control, test administration rules and neuropsychological testing techniques. It also covered potential pitfalls of administering cognitive tests in low-resource settings like Kenya and the sensitivities involved in administering tests to ensure reliability and validity. Working with local assessors who were knowledgeable of the local language and culture as well as a wealth of experience administering neurocognitive and psychological assessments in the same study setting enabled a smooth training experience, and endorsement and acceptance of the study by the local community. Several pre-tests and using role-play helped ensure assessor scoring was consistent, accurate and standardized. Through facilitated debriefing sessions, discrepancies were identified and addressed, fostering a shared understanding of scoring criteria and interpretation.

All measures not previously adapted to the local language (Swahili) underwent adaptation procedures that comprised forward translation, forward translation review, back translation, back translation review, harmonization by a panel of experts (comprising a research psychologist, global mental health practitioner, and 4 research assistants from the KEMRI-Wellcome Trust Research Program (KWTRP) who had extensive experience administering neuropsychological assessments in the local setting), pilot testing, pilot testing review and proofreading (Abubakar and Van De Vijver, 2017). The expert panel members were conveniently selected from the Neurosciences department at KWTRP based on their subject expertise, experience in neuropsychological assessments and knowledge of the local culture. Where applicable (e.g., OCSPlus tool), an international representative from the team of tool developers was included in the harmonization process. We conducted two rounds of pilot testing and participants provided feedback regarding item relevancy, word clarity and ambiguities, content, cultural nuances, flow of questions, response options, and administration process and logistics which was then synthesized in coming up with the revised version.

#### Sociodemographic information

We captured sociodemographic characteristics, including participants’ age, sex, marital status, educational level, household size, household income, and living arrangements. Additional information included individual/family ownership of disposable assets (for asset index calculation as a proxy for socioeconomic status). Participants also gave information about food security, social support, caregiving responsibilities and whether they were utilizing traditional medicine.

#### General health data

This information included participants’ anthropometric details (e.g., height, weight, blood pressure, waist, and hip circumference), sexual activity, levels of physical activity, number of medications one was using, self-reported comorbidities, past medical history, and common complaints, e.g., pain, fatigue, sleeping problems, visual and hearing problems.

For older adults living with HIV, we also asked HIV-specific questions, e.g., disclosure of HIV status, access to HIV services, cART regimen, past HIV medical history such as regimen interruption, and prolonged illness after HIV diagnosis. Information regarding the current ART regimen and overall ART duration were extracted from their clinic medical records. Additionally, we collected 10 mL of venous blood samples from the OALWH for viral load testing.

#### Psychosocial data

Psychosocial variables included HIV-related stigma, functional disability, ageism, and loneliness. These constructs were assessed using interviewer-administered Likert scales: the brief 12-item HIV stigma scale (Reinius et al., 2017), the 12-item World Health Organization Disability Assessment Schedule 2 (Üstün et al., 2010), the UCLA (University of California, Los Angeles) 8-item loneliness scale (Hays and DiMatteo, 1987), and the 20-item ageism survey (Palmore, 2001). For each scale, a higher score translates into greater impairment.

#### Neuropsychological evaluation

We used various measures to evaluate cognitive function, including:

International HIV Dementia Scale (IHDS). The IHDS is a conventional brief 3-min screening tool comprising tasks that assess verbal memory, motor and psychomotor performance (Mwangala et al., 2018; Sacktor et al., 2005). The highest possible score is 12, with higher scores indicating better cognitive performance. A score of less than 10 shows a reasonable sensitivity (64–74%) and specificity (55–66%) in identifying moderate to severe HIV-associated neurocognitive impairment (Haddow et al., 2013).

Ravens Standard Progressive Matrices (RSPM) (Raven et al., 1977): The RSPM is a non-verbal standardized test of general intelligence (non-verbal intelligence) comprising five series with 12 items. The measure has previously been used in Kilifi, yielding good psychometric properties (Nyongesa et al., 2018). The maximum possible score is 60, with a higher score indicating better cognitive performance.

Oxford Cognitive Screen Plus (OCSPlus) (Demeyere et al., 2021): OCSPlus is a tablet-based cognitive assessment tool suitable for low-literacy, low-income settings. OCSPlus assesses distinct cognitive domains, such as language, memory, attention, and executive functioning. Participants’ performance and timing are automatically recorded and scored. Initial validation in rural South Africa yielded excellent construct and external validity (Humphreys et al., 2017). The adapted Swahili version was used in this study. Briefly, the memory domain included: (a) an orientation task assessing orientation in time and space, (b) a 5-word immediate recall task where the participant has two stages of encoding (encoding 1 and 2) – the participant is given a list of 5 words to remember and asked to recall the items immediately; then regardless of performance the participant is presented with the list again and asked for immediate recall a final time; (c) delayed recall (4 to 5-min delay) that assesses the recall of the 5 encoded words; (d) incidental memory that assesses incidental recognition of items and tasks which the participant experienced earlier in the assessment. The language domain was assessed by a picture naming task with four target pictures and a test of semantic knowledge (four objects). The executive function domain assessment consisted of three versions of non-verbal trails task where participants were required to connect shapes using different rules: first circles, then squares, and then alternating between circles and squares. The trails items were all treated as components of the same test. Processing speed was calculated as the sum of time taken on both versions of trails baseline tasks (circles or squares) divided by proportional accuracy. The attention domain was measured using a cancellation task. The participant selects drawings of fruit among drawings of common fruits and vegetables immediately followed with an invisible version of the same display. Demeyere et al. (2021) provides a comprehensive description of the OCSPlus development and validation.

OCSPlus was administered by two diploma-level research assistants who have several years’ experience conducting psychological assessments at the KWTRP. The two assessors underwent 1 week of training at the beginning (and a subsequent refresher at mid-project) on how to conduct neurocognitive assessments including the OCSPlus. The training was led by a master’s level mental health researcher with guidance from the tool developers. Following training, the team conducted pilot activities with adults in the community. To evaluate the acceptability and feasibility of the OCSPlus tool, clients and test administrators responded to post-test survey questions (both closed and open-ended questions) about their experience using the tool. Clients were asked to indicate their ease of using the tool, e.g., their interactions with the tablet, and the degree of difficulty of the battery of tests. Test administrators provided feedback through an open-ended questionnaire in addition to debriefing sessions. Responses included noting how easy/difficult it was for clients to understand instructions, and as well as their own experience with the tool. Test–retest participants were randomly selected from the pool of assessed participants (1 month after the initial assessment) by the data manager and recontacted to come for the follow up assessments at the KWTRP. The test–retest assessors were the same at both timepoints. The choice of IHDS and RSPM as comparator instruments to OCSPlus in the present study was informed by the tool’s prior adaptation and use in the current study setting. We also considered brief measures to avoid making participants very tired.

### Data analysis

All analyses were conducted in STATA version 15.0 (StataCorp LP). We used descriptive statistics (chi-square tests and student t-tests) to summarize sample characteristics and where appropriate, we used their non-parametric equivalents. Test–retest reliability was assessed using intra-class correlations (two-way mixed model). To examine convergent validity between the OCSPlus tests and the conventional tests, Pearson correlation coefficients were computed for each OCSPlus test. We aimed for correlations above 0.3 to demarcate convergence (Rotenberg et al., 2020) but also cautiously interpreted correlations >0.19 (Demeyere et al., 2021). Correlations between 0.19 and 0.29 were considered weak but still suggestive of convergence. This approach is consistent with earlier validation of the OCSPlus (Demeyere et al., 2021). We used the Wilcoxon rank-sum test to compare cognitive performance between older adults living with HIV and their uninfected peers. The multiple comparisons likely gives rise to familywise Type I error that is controlled in the current project by using the false discovery rate (FDR) method (Benjamini and Hochberg, 1995). This method is less conservative and has greater efficiency when compared to other approaches like the Bonferroni correction (Glickman et al., 2014). Specifically, we conducted 17 separate Wilcoxon rank-sum tests on 17 different cognitive sub-tests. The FDR formula is given by *p_i_* = *α* * i / n, where *p_i_* represents the adjusted *p* value for the *i^th^* dependent variable with the *i^th^* smallest pre-adjusted p value. Here, we set α = 0.05 as the pre-set *p*-value, *n =* 17, and i takes values 1, 2, 3, 4, 5…17 because of the 17 separate tests. All the *p-*values reported in Table 1 have undergone this adjustment. The eight lowest *p-*values were less than their corresponding FDR values; thus, significant at the 0.05 false discovery rate and the others were not significant. Finally, we conducted multiple linear regressions to examine the association between OCSPlus raw scores and biopsychosocial variables among the participants. To do this, univariable linear regression analyses were conducted to identify factors associated with OCSPlus raw scores and all factors having a p-value of ≤ 0.20 (Katz, 2011) were then entered into a multivariable linear regression model to examine the independent correlates through a back-ward elimination process (Royston et al., 2009). In all the multivariable models, we performed collinearity diagnostics using STATA’s ‘collin’ syntax and no multicollinearity problems were identified based on an interpretation of the variance inflation factor. In all cases, the data was verified for skewness using histograms and calculating skewness coefficients to determine need for data transformation. Statistical significance was considered at *p <* 0.05.

**Table 1:** Cognitive profile on conventional and OCSPlus cognitive tests, according to HIV status (*n =* 440).

Domain	Test	Older adults living with HIV (*n =* 257)	Older adults without HIV (*n =* 183)	*P*-value
		Median (IQR)	Median (IQR)	
OCSPlus sub-tests				
Language *(A higher score is better cognitive performance)*	*Picture naming task*	4.0 (4.0–4.0)	4.0 (4.0–4.0)	<0.001
	*Semantics task*	2.0 (2.0–3.0)	2.0 (2.0–3.0)	0.1
Memory *(A higher score is better cognitive performance)*	*Orientation task*	4.0 (2.0–4.0)	2.0 (1.0–3.0)	**<0.001**
	*Word encoding task 1*	3.0 (2.0–4.0)	3.0 (2.0–4.0)	0.3
	*Word encoding task 2*	4.0 (4.0–5.0)	4.0 (3.0–5.0)	**0.02**
	*Word recall - free score*	2.0 (1.0–3.0)	2.0 (1.0–3.0)	**0.001**
	*Word recall - total score*	3.0 (3.0–4.0)	3.0 (2.0–4.0)	0.1
	Incidental - *memory*	3.0 (2.0–3.0)	3.0 (2.0–3.0)	0.5
Executive functioning	Trails - *Baseline trails*	9.0 (5.0–12.0)	8.0 (4.0–12.0)	0.3
	Trails - *Executive score*	0.6 (0.3–1.0)	1.0 (0.5–1.4)	**<0.001**
Processing speed *(A lower score is better cognitive performance)*	Trails - *processing speed score (milliseconds)*	94157.5 (57830.5–191499.6)	135350.3 (68265.2–239052.5)	**0.008**
Attention *(A higher score is better cognitive performance for taps on targets, and the opposite is true for taps on distractors)*	Selection visible task - *Number of unique taps on targets*	27.0 (19.0–29.0)	26.0 (19.0–29.0)	0.9
	Selection visible task - *Number of unique taps on distractors*	0 (0–6)	1.0 (0–10.0)	0.04
	Selection invisible task - *Number of unique taps on targets*	19.0 (10.0–26)	20.0 (14.0–25.0)	0.6
	Selection invisible task - *Number of unique taps on distractors*	0 (0–2.0)	0 (0–7.0)	0.07
Conventional measures				
Non-verbal intelligence	Ravens standard progressive matrices	17.0 (14–22)	15.0 (13.0–18.0)	**0.001**
verbal memory, motor, and psychomotor performance *(A higher score is a better cognitive performance)*	International HIV Dementia Scale	8.0 (6.0–9.0)	8.0 (7.0–9.0)	**<0.001**

Cognitive domains with significant differences at the 0.05 false discovery rate are indicated with bolded *p-*values. All the values are based on the Mann–Whitney U test.

## Results

### Sample characteristics

The final sample comprised 440 older adults (58% living with HIV), with a median age of 59 (range 54–64) years and a participant response rate of 90%. Female participants slightly outnumbered males comprising 58.6% of the sample. The majority of the participants (63.2%) had formal education, with an average of 4.8 years of formal schooling, 65.5% were unemployed, and 81.6% were living in multigenerational households. All the adults living with HIV were on HIV treatment with a mean duration of HIV treatment of 11.4 years (SD = 4.3). Most of the OALWH had disclosed their HIV status (95.3%) and were on first-line cART treatment (90%). Most (98%) of the OALWH were virally suppressed. Further details of the sample characteristics have been reported in Mwangala et al. (2022).

### Preliminary psychometrics

#### Acceptability of the OCSPlus tool

Ninety-four percent (*n =* 407) of participants reported never having used a computer. Only 22 % (*n =* 88) had used a smartphone previously. Only 2 % (*n =* 10) reported using a tablet before. Fifty-three percent of the sample reported that the study tablet was “very easy” to “somewhat easy” to use, while the rest (47%) felt the study tablet was “somewhat difficult” to “very difficult to use.”

Table 2 gives respondents’ perceived degree of difficulty or ease on the different OCSPlus subtests. Trail making test was reported to be the most challenging test by 96 (22%) of the respondents. On the other hand, the selection visible OCSPlus task was considered the easiest by 349 (79%) of the participants. On average, study participants took 17 min to complete the OCSPlus sub-tasks. Further details on the different OCSPlus subtasks are highlighted in Table 2.

**Table 2:** Perceived degree of difficulty or ease on various OCSPlus tests from respondents.

OCSPlus subtest	Respondents who reported that the test was difficult	Respondents who reported that the test was easy
Picture naming	35 (8%)	270 (61%)
Semantics	39 (9%)	267 (61%)
Orientation	31 (7%)	268 (61%)
Word encoding 1	68 (15%)	239 (54%)
Word encoding 2	59 (13%)	242 (55%)
Trail making	96 (22%)	244 (55%)
Delayed word recall	71 (16%)	236 (54%)
Incidental memory	30 (7%)	249 (57%)
Selection visible	48 (11%)	349 (79%)
Selection invisible	67 (15%)	306 (70%)

Feedback from the test administrators was generally positive. Administrators rarely encountered any technical problems when administering the test. In three instances, the tablet froze; however, restarting the tablet was able to fix the problem. Other reported challenges included eyesight difficulties and hand tremors, which were experienced by 8 participants.

#### Reliability

A group of 46 older adults living with HIV were re-tested on the OCSPlus tool on average 30 days after the initial test. The time between test administrations (1 month) was deemed long-enough to minimize practice effects. Intra-class correlations were computed to examine test–retest reliability for the different OCSPlus subtasks highlighted in Table 3. Reliability coefficients were generally of acceptable quality, ranging from 0.51 to 0.79.

**Table 3:** Intra class correlations for the different OCSPlus subtasks.

Measure	Reliability coefficients (95% CI)
Picture naming	0.78 (0.61, 0.83)
Semantics	0.68 (0.46, 0.77)
Orientation	0.79 (0.65, 0.88)
Word encoding 1	0.60 (0.37, 0.76)
Word encoding 2	0.53 (0.28, 0.71)
Delayed word recall	0.51 (0.26, 0.70)
Trails circles	0.57 (0.33, 0.73)
Trails squares	0.51 (0.26, 0.70)
Trails baseline	0.68 (0.48, 0.81)
Cancellation	0.68 (0.47, 0.80)

We also calculated Cronbach alphas for the different OCSPlus domains; most alpha values were low (Table 4). However, performance on these items was stable over time.

**Table 4:** Cronbach alphas for some of the OCSPlus subtasks.

Measure	Cronbach alphas (95% CI)
Picture naming	0.58 (0.47, 0.68)
Semantics	0.67 (0.59, 0.75)
Orientation	0.67 (0.62, 0.72)
Word encoding	0.57 (0.53, 0.62)
Word recall	0.68 (0.57, 0.73)

#### Convergent validity

To examine convergent validity between the OCSPlus tests and the conventional tests, Pearson correlation coefficients were computed for each OCSPlus test. Table 5 displays the correlation matrix between OCSPlus and the conventional tests. Generally, statistically significant correlations between the OCSPlus and conventional tests were observed for most tests.

**Table 5:** Correlations between OCSPlus and the conventional tests (*n =* 440).

Domain	Ravens total score	IHDS total score
OCSPlus subtasks		
Picture naming	0.1	0.3**
Semantics	0.3**	0.2**
Orientation	0.5**	0.2**
Word recall total score	0.3**	0.3**
Incidental memory	0.2**	0.2**
Trails circle	0.5**	0.2**
Trails squares	0.5**	0.3**
Trails mixed	0.2**	0.3**
Selection visible targets	0.3**	0.3**
Selection invisible targets	0.3**	0.4**

**p < 0.01.

#### Cognitive performance on conventional and OCSPlus measures

Table 1 gives the median scores for the OCSPlus sub-tasks and the conventional cognitive measures (RSPM and the IHDS). OALWH presented significantly lower scores on language (picture naming subtask), executive function (trails), and the conventional screening measure (IHDS) compared to their uninfected counterparts. However, OALWH performed significantly better in memory (orientation, word encoding and free recall subtasks), non-verbal intelligence and processing speed. There were no differences in attention.

#### Association of OCSPlus raw scores with biopsychosocial variables

Table 6 shows biopsychosocial variables predicting OCSPlus raw scores among older adults living with HIV. The most consistent finding in this group was that education was a significant independent positive predictor of OCSPlus mean scores across all cognitive domains. Female sex was significantly independently associated (negatively) with all but one (attention) of the five OCSPlus cognitive domains. Moreover, as expected, age was significantly independently associated (negatively) with memory, executive functioning, and attention scores. Asset index (a proxy measure of socioeconomic status) was positively independently associated with memory scores, while food insecurity was negatively related with the same domain. Monthly household income and being sexually active were positively independently associated with executive scores, while self-reported urinary incontinence was negatively independently associated with the same domain. Doing light physical activities was positively independently associated with language scores, while sleeping difficulties were negatively independently associated with the same domain. Self-reported hearing problems, ART regimen change and seeking services of traditional healers were negatively independently associated with attention scores. Ageism was negatively independently associated with executive and attention scores. Caring for a sick family member was positively independently associated with language and attention scores. An increasing number of dependents was negatively independently associated with language, executive and processing speed scores. Being on third-line HIV treatment was negatively independently associated with language and memory scores.

**Table 6:** Multiple linear regression model showing the association of OCSPlus raw scores with biopsychosocial variables among OALWH.

Independent variables	β-coefficient (95% CI) of cognitive domains as dependent variables											
	Language		Memory				Executive			Processing speed	Attention	
	Picture naming	Semantics	Orientation	First immediate word recall	Delayed word recall	Incidental memory	Trails, circles	Trails, squares	Trails, mixed	Trails – processing speed score	Selection, visible	Selection, invisible
Sociodemographic characteristics												
Female sex (ref: male)	−0.1 (−0.2; 0.04)	−0.3*** (−0.5; −0.2)	−0.5*** (−0.7; −0.2)	−0.1 (−0.4; 0.2)	−0.2 (−0.5; 0.1)	−0.03 (−0.2; 0.2)	−0.8** (−1.4; −0.2)	−0.7** (−1.3; −0.2.)	1.2 (−0.1; 2.4)	72298.4** (1463.9; 143132.9)	−0.2 (−2.4; 2.1)	−1.3 (−3.3; 0.6)
**Age** (ref: 50-59 years)												
60–69 years	−0.04 (−0.2; 0.1)	0.02 (−0.2; 0.2)	−0.01 (−0.2; 0.2)	−0.1 (−0.4; 0.2)	−0.6*** (−0.9–0.3)	0.1 (−0.1; 0.3)	−0.1 (−0.7; 0.5)	−0.6** (−1.1–0.1)	0.6 (−0.5; 1.7)	14190.6 (−54615.9; 82997.1)	−1.6 (−3.6; 0.4)	−2.2** (−4.2; −0.3)
≥70 years	−0.2 (−0.5; 0.04)	0.03 (−0.3; 0.4)	−0.2 (−0.6; 0.3)	−0.1 (−0.7; 0.4)	−1.0*** (−1.5–0.5)	−0.3 (−0.7; 0.05)	−0.8 (−2.0; 0.4)	−1.3** (−2.3–0.3)	1.6 (−0.5; 3.8)	74949.1 (−64132.2; 214030.4)	−6.0*** (−10.1; −1.9)	−6.0*** (−9.9; −2.1)
**Education** (ref: none)												
Primary level (upto completion)	−0.2 (−0.3; 0.0)	0.2 (−0.01; 0.4)	1.0*** (0.8; 1.3)	0.4** (0.03; 0.7)	0.6*** (0.3; 0.9)	0.4*** (0.2; 0.6)	1.9*** (1.2; 2.6)	0.9*** (0.3; 1.6)	1.6** (0.4; 2.8)	−149863.4*** (−230170.3; −69556.5)	1.7 (−0.7; 4.0)	3.0** (0.7; 5.4)
Secondary level (upto completion)	−0.1 (−0.3; 0.1)	0.3** (0.001; 0.5)	1.2*** (0.9; 1.6)	0.5** (0.03; 0.9)	0.5*** (0.1; 0.9)	0.5*** (0.2; 0.8)	2.9*** (2.0; 3.8)	1.9*** (1.1; 2.7)	2.5*** (1.0; 4.1)	−200253.8*** (−302309.7; −98198.0)	4.8*** (1.8; 7.8)	4.3*** (1.3; 7.3)
Tertiary level (upto completion)	−0.3 (−0.5; 0.0)	0.4** (0.04; 0.8)	1.1*** (0.6; 1.6)	0.8*** (0.2; 1.4)	1.1*** (0.1; 0.7)	0.4** (0.03; 0.8)	2.8*** (1.6; 4.0)	1.5** (0.4; 2.6)	2.6** (0.4; 4.8)	−244202.9*** (−387041.2; −101364.5)	0.8 (−3.2; 4.7)	3.9 (−0.1; 7.9)
Asset index score	–	–	0.1*** (0.1; 0.2)	0.1*** (0.04; 0.2)	–	–	–	–	–	–	–	–
Monthly household income >10,000 Ksh (ref: ≤10,000 Ksh)	–	–	–	–	–	–	–	1.1*** (0.4; 1.8)	2.5*** (1.2; 3.9)	–	–	–
Health-related characteristics												
Food insecurity in the past week (ref: never)												
Sometimes	–	–	–	–	−0.3** (−0.6; 0.03)	–	–	–	–	−19886.4 (−93226.0; 53453.2)	–	–
Most of the time	–	–	–	–	−0.2 (−0.6; 0.2)	–	–	–	–	140935.2*** (27179.5; 254691.0)	–	–
Sexual intercourse (ref: no)	–	–	–	–	–	–	–	–	1.8*** (0.6; 2.9)	–	2.0 (−0.3; 4.2)	
Days spent doing light activities in the past week	0.1*** (0.02; 0.1)	–	–	–	–	–	–	–	–	–	–	–
Self-reported hearing (ref: good)	–	–	–	–	–	–	–	–	–	–	−2.9** (−5.3; −0.5)	–
Sleeping difficulties in the past month (ref: none)												
Sometimes	−0.1** (−0.3; 0.03)	–	–	–	–	–	–	–	–		–	–
Most of the time	−0.2 (−0.4; 0.01)	–	–	–	–	–	–	–	–		–	–
Access to social support (ref: none)	0.2 (−0.02; 0.5)	–	–	–	–	–	–	–	–		–	–
Ageism scores	–	–	–	–	–	–	–	−0.1*** (−0.1; 0.03)	–		–	−0.2*** (−0.4; −0.1)
Caring for a sick family member (ref: no)	0.3*** (0.2; 0.5)	–	–	−0.2 (−0.5; 0.1)	–	–	–	–	–		8.6*** (6.4; 10.7)	8.4*** (5.9; 10.9)
Number of dependents	–	−0.04** (−0.1; 0.01)	–	–	–	–	−0.1** (−0.2; 0.04)	−0.1*** (−0.2; 0.03)	–	16146.7*** (4199.0; 28094.5)	–	–
Number of drugs the client currently uses	–	–	–	–	–	–	–	–	–	33344.3*** (6694.2; 59994.4)	–	–
cART regimen (ref: 1^st^ line)												
2^nd^ line	–	0.04 (−0.3; 0.3)	0.3 (−0.03; 0.7)	–	–	0.2 (−0.1; 0.5)	–	–	–	–	–	–
3^rd^ line	–	−1.7** (−3.1; 0.4)	−0.8 (−2.4; 0.9)	–	–	−1.6** (−3.0; −0.1)	–	–	–	–	–	–
History of cART change (ref: no)	–	–	–	–	–	–	–	–	–	–	–	−4.6*** (−6.8; −2.4)
History of frequent body pain (ref: no)	–	–	–	–	0.4*** (0.1; 0.7)	0.3** (0.1; 0.5)	–	–	–	–	–	–
History of stomach upsets	–	–	–	–	–	–	–	–	–	–	–	−2.6 (−7.3; 2.1)
Mid-upper arm circumference	–	–	–	–	–	–	0.1** (0.01; 0.1)	–	–	–	–	–
Incontinence - urinary (ref: no)	–	–	–	–	–	–	–	–	−2.2** (−3.9; 0.4)	–	–	–
Seeking services of traditional healers (ref: no)	–	–	–	–	–	–	–	–	–	–	−4.5** (−8.0; −1.0)	–
n, final model	246	249	253	247	252	249	250	254	250	247	248	248
R-squared, final model	28.1%	14.2%	47.5%	17.3%	28.3%	14.7%	30.6%	32.1%	16.8%	21.3%	31.5%	41.9%

**p < 0.05; ***p < 0.01; Predictors with no information on beta values were not unique significant predictors in the simultaneous multi-linear regression.

Table 7 shows the biopsychosocial variables associated with OCSPlus raw scores among HIV uninfected older adults. Among HIV uninfected older adults, education was significantly independently associated (positively) with memory, executive and processing speed domains. Female sex was significantly independently associated (negatively) with language and memory scores. Age was negatively independently associated with language, processing speed and attention scores. Asset index was positively independently associated with language scores. Increasing number of dependents was negatively associated with memory scores. Self-reported hearing problems were negatively independently associated with executive scores. A history of tuberculosis was negatively independently associated with attention scores. Food insecurity was negatively independently associated with attention scores. Being overweight and frequent fatigue were negatively associated with processing speed scores.

**Table 7:** Multiple linear regression model showing the association of OCSPlus raw scores with biopsychosocial variables among HIV uninfected older adults.

Independent variables	β-coefficient (95% CI) of cognitive domains as dependent variables										
	Language	Memory				Executive			Processing speed	Attention	
	Semantics	Orientation	First immediate word recall	Delayed word recall	Incidental	Trails, circles	Trails, squares	Trails, mixed	Trails – processing speed score	Selection, visible	Selection, invisible
Sociodemographic characteristics											
Female sex (ref: male)	−0.6*** (−0.8; −0.3)	−0.4*** (−0.8; −0.1)	0.2 (−0.2; 0.5)	−0.2 (−0.6; 0.2)	−0.1 (−0.4; 0.1)	−0.3 (−1.1; 0.5)	−0.2 (−0.9; 0.6)	0.4 (−1.2; 2.0)	18254.2 (−76428.8; 112937.2)	−0.7 (−3.1; 1.7)	−0.5 (−2.9; 1.8)
Age (ref: 50–59 years)											
60–69 years	−0.03 (−0.3; 0.2)	0.1 (−0.3; 0.4)	−0.01 (−0.4; 0.3)	−0.2 (−0.6; 0.2)	−0.1 (−0.4; 0.2)	−0.6 (−1.3; 0.2)	−0.5 (−1.2; 0.2)	0.5 (−1.0; 2.0)	89259.2** (4521.5; 173996.8)	0.1 (−2.2; 2.3)	−1.1 (−3.3; 1.1)
≥70 years	−0.4** (−0.7; 0.02)	0.2 (−0.3; 0.7)	0.1 (−0.4; 0.6)	−0.5 (−1.1; 0.1)	−0.3 (−0.7; 0.2)	0.3 (−0.8; 1.3)	−0.6 (−1.7; 0.5)	0.1 (−2.2; 2.3)	154362.1** (28638.9; 280085.3)	−2.0 (−5.4; 1.3)	−4.2** (−7.6; −0.8)
Education (ref: none)											
Primary level	−0.05 (−0.3; 0.2)	1.1*** (0.8; 1.5)	0.6*** (0.2; 1.0)	0.5** (0.04; 0.9)	0.2 (−0.1; 0.5)	1.0** (0.2; 1.9)	1.4*** (0.6; 2.2)	2.5*** (0.8; 4.2)	−119893.2** (−213755.6; −26030.7)	1.3 (−1.3; 3.8)	
Secondary level	0.1 (−0.3; 0.5)	1.8*** (1.2; 2.3)	0.8*** (0.3; 1.3)	1.0*** (0.4; 1.6)	0.3 (−0.1; 0.8)	3.1*** (1.9; 4.3)	2.8*** (1.5; 4.0)	3.7*** (1.3; 6.1)	−244097.7*** (−378665.5; 109529.9)	2.4 (−1.4; 6.2)	2.2 (−0.3; 4.7)
Tertiary level	0.02 (−0.6; 0.6)	0.8 (−0.1; 1.8)	0.2 (−0.8; 1.2)	0.5 (−0.5; 1.5)	−0.1 (−0.8; 0.6)	2.6*** (0.7; 4.5)	2.2** (0.3; 4.2)	3.9** (0.02; 7.8)	−181,528 (−398055.6; 34999.7)	3.0 (−3.0; 9.0)	3.4 (−0.1; 7.0)
Asset index score	0.1*** (0.04; 0.2)	–	–	–	–	−0.1 (−0.4; 0.2)	−0.02 (−0.3; 0.3)	–	–	0.6 (−0.3; 1.6)	4.2 (−2.3; 10.7)
Employment (ref: none)											
Employed	–	0.1 (−0.3; 0.5)	0.1 (−0.3; 0.5)	–	–	–	–		–		
Retired	–	0.5 (−0.1; 1.0)	0.6 (−0.03; 1.1)	–	–	–	–		–		
Number of dependents	–	–	–	−0.1** (−0.2;-0.02)	–	–	–		–		
Monthly household income >10,000 Ksh (ref: ≤10,000 Ksh)	–	–	–	–	–	–	–	–	0.3 (−0.05; 0.6)		
Health-related characteristics											
Self-reported hearing (ref: good)	–	–	–	–	–	–	–	−2.0** (−3.8; −0.2)			
Past history of TB (ref: no)	–	–	–	–	–	–	–	–	–	−15.9** (−29.8; −2.0)	–
Food insecurity in the past week (ref: never)											
Sometimes	–	–	–	–	–	–	–	–	79050.4 (−9371.8; 167472.6)	–	−2.5** (−4.8; −0.1)
Most of the time	–	–	–	–	–	–	–	–	−446.6 (−231369.1; 230475.8)	–	−6.6 (−14.9; 1.7)
BMI Categories (ref: normal)											
Underweight	–	–	–	–	–	–	–	–	51386.0 (−88180.5; 190952.4)	–	–
Overweight	–	–	–	–	–	–	–	–	126147.0 *** (34067.3; 218226.7)	–	–
Obese	–	–	–	–	–	–	–	–	5823.6 (−119896.6; 131543.8)	–	–
Frequent fatigue	–	–	–	–	–	–	–	–	176293.6 *** (55624.7; 296962.4)	–	–
n, final model	179	182	175	181	176	181	181	181	181	181	180
R-squared, final model	21.9%	44.8%	13.4%	15.0%	5.5%	20.6%	19.3%	12.2%	27.4%	9.3%	13.8%

**p < 0.05; ***p < 0.01; Predictors with no information on beta values were not unique significant predictors in the simultaneous multi-linear regression.

## Discussion

Although HIV-associated neurocognitive impairments are mostly mild in patients successfully treated with modern ART, ageing is likely to exacerbate severe forms of impairments with greater impacts on activities of daily living and health-related quality of life. Unfortunately, valid and reliable screening tools for these impairments are lacking in many parts of SSA, including Kenya. To bridge this gap, our study provides preliminary evidence for the acceptability, reliability (internal consistency and test–retest), and validity (convergent and external validity) of the Oxford Cognitive Screen Plus (OCSPlus) tool, a brief, tablet-based, domain-specific cognitive assessment designed for low-literacy settings in a hospital sample of adults ageing with HIV (≥50 years) and their uninfected peers at the Kenyan coast. Additionally, our study documents the cognitive performance of these adults (by HIV status) and reports the factors associated with their cognitive performance. The current paper and data present the initial steps toward building valid and reliable neurocognitive assessment tools for ageing populations in Kenya and similar contexts, and open opportunities for further research, e.g., largescale validation of the tool to further confirm its validity and reliability and generate normative scores before testing the tool’s utility in clinical settings.

Our findings demonstrate the feasibility of layperson-administered older adults cognitive testing with the OCSPlus in a low-literacy Kenyan setting. All the administered tasks had >90% valid data. There was also a high level of acceptability of OCSPlus by the participants. Performance on most of the OCSPlus subtasks correlated well with the conventional cognitive tests, although some of the correlations were relatively low. OCSPlus was also found to have good test–retest reliability. Regarding cognitive performance, OALWH had significantly lower scores in language (picture naming task), executive function and one of the conventional measures (IHDS) compared to their uninfected peers. However, they (OALWH) performed better in the memory domain (orientation, word encoding and word recall tasks), non-verbal intelligence and processing speed. Cognitive performance among OALWH (on OCSPlus domains) was predicted by a host of biopsychosocial variables ranging from sociodemographic factors (female sex, age, educational level, asset index, monthly household income, number of dependants), psychosocial factors (food insecurity, sleeping difficulties, ageism, physical activity, sexual activity), biomedical (self-reported urinary incontinence, hearing problems) and treatment-related factors (ART regimen change, being on third-line HIV treatment, and seeking the services of traditional healers). All the observed associations were in the hypothesized direction. Similar associations were also seen among HIV uninfected older adults. Our findings extend the evidence on cognitive screening instruments among older adults in the region and pave the way for further research on the utility and application of the OCSPlus tool in the region and similar settings.

To the best of our knowledge, OCSPlus has been normed and validated in healthy ageing cohorts in the UK and Germany (Demeyere et al., 2021), validated among patients with amnestic mild cognitive impairment in Germany (Hübner et al., 2020) and has been used in epidemiological research in a low literacy setting in South Africa (Humphreys et al., 2017) and recently validated for use in stroke survivors in the UK (Webb et al., 2022; Roberts et al., 2024). The current study is the second one in Africa, besides the South African study, to adapt and validate the OCSPlus for use among adults ageing with HIV in the region. The initial validation in South Africa was conducted in a population of older adults (aged at least 40 years) in rural South Africa against standard questionnaires (for construct validity) and in relation to associations with several variables, including physical and mental health, age, education, and alcohol consumption (for external validity) (Humphreys et al., 2017). The authors reported high task compliance and good construct and external validity of the OCSPlus in their large, low-education, low-income population sample from the rural South African community of Agincourt. Unlike the South African study (which included 1,402 adults), the OCSPlus in our study was validated among older adults ≥50 years, and our sample was smaller (440 older adults), comprising OALWH in routine HIV care and HIV-uninfected older adults from the surrounding communities. Generally, our findings confirm the validity of the OCSPlus tool among low-literacy, low-income populations in SSA. Our study also provides preliminary evidence of the tool’s reliability in Kenya with acceptable test–retest results. Only internal consistency and test–retest are reported in this study. Additional studies are needed to explore other aspects of reliability. A variant of the OCSPlus tool known as the Oxford Cognitive Screen: Executive Function (OCS-EF) has also been validated in SSA, albeit among adolescent females in rural South Africa. The results indicated that the tool can be administered by trained lay people and is valid for assessing cognition at scale among adolescents (Rowe et al., 2021). Outside SSA, the OCSPlus has also been validated among 320 neurologically healthy older adults from a pooled English and German normative sample (Demeyere et al., 2021). OCSPlus subtasks were found to have good divergent validity. Additionally, performance on several OCSPlus subtasks correlated with performance on analogous standard measures, though some of the convergent validity was relatively low. OCSPlus was also found to have good test–retest reliability despite the wide-ranging test–retest intervals.

Regarding convergent validity, the range of correlation coefficients reported in this study (0.2–0.5) represents an acceptable level of convergent validity in reference to the values reported in the original OCSPlus tool (Demeyere et al., 2021). The relatively low convergent validity may be attributed to the unique ability of this test to measure neuropsychological functions that were indistinguishable with existing cognitive-screening tools. Alternatively, this could be because of the conventional tools used in this study – which utilized a global neurocognitive score, and not individual cognitive domains scores similar to OCSPlus. In terms of the size of the convergent correlations, the majority were at or above an acceptable level of convergence observed in validations of other commonly used screens, that is, >0.20 (Demeyere et al., 2021). While the performance on OCSPlus subtasks and conventional tests are significantly associated, these tests are not identical or have very few lower-range scores to give perfect estimates. We recommend additional studies to further explore these aspects of validity.

In our second objective, OALWH presented with significantly lower cognitive scores on language (picture naming subscale) and executive function and on the IHDS. However, the same adults performed significantly better in memory domain (orientation, word encoding, and word recall subscales), non-verbal intelligence and processing speed. Our findings are partly different from those of a population-based cross-sectional study of older adults in rural South Africa (Asiimwe et al., 2020). When using a conventional battery (assessing orientation, word recall, and numeracy skills), the authors observed significantly higher cognitive scores among PLWH. Nonetheless, when cognitive function was evaluated using OCSPlus, the authors found no significant differences in cognitive function according to HIV and ART status. The discrepancy in findings may perhaps be due to differences in the samples of OALWH studied (population-based vs. hospital sample), tools used (conventional ones), participants’ age (≥50 years in our study vs. ≥ 40 years in the South African study) and other contextual differences. The South African study used summative cognitive scores (for both the conventional and OCSPlus test scores) compared to the Kenyan study, which used domain-specific test scores in the OCSPlus. Our findings of lower cognitive scores (language, executive function and IHDS) among OALWH is consistent with previous findings which tend to show lower cognitive scores among people living with HIV compared to their uninfected peers (Sheppard et al., 2015). Our observations of better cognitive scores among OALWH (memory, non-verbal intelligence and processing speed) add to a growing literature in SSA showing that people living with HIV have similar or better health outcomes compared to their uninfected peers (Mwangala et al., 2021) potentially due to, e.g., differential access to healthcare, and selective survival among people living with HIV. The OCSPlus tool assesses several domains of cognitive function, and some of these domains may be especially sensitive to HIV-related brain changes. Our results also highlight that different measures may produce different conclusions, and that the cultural validation of cognitive screens is very important in cognitive assessment in HIV populations.

Our study also identified several variables associated with cognitive performance (across OCSPlus subtasks). These factors ranged from behavioral and lifestyle factors (physical activity, sleeping difficulties, obesity, and sexual activity), sociodemographic factors (age, sex, educational status, household income, household size and asset index), medical or treatment factors (self-reported urinary incontinence, hearing problems, history of TB, seeking services of traditional healers, ART regimen change, being on 3^rd^ line ART treatment) and psychosocial factors including ageism and food insecurity. The observed associations were in the expected direction, consistent with previous literature. Notably, many of these factors are modifiable, hold great promise for promoting cognitive health, and may lend themselves to developing effective interventions in this population. Our findings, however, do not infer causality; further research with suitable study designs, e.g., longitudinal studies, is needed to understand the results further. Similar findings on some of these variables have been reported elsewhere in West Africa (Bernard et al., 2023; Bernard et al., 2021) and South Africa (Kobayashi et al., 2019).

### Implications

By 2040, about 70% of the 80 million individuals with dementia will reside in LMICs, where many older adults have little formal education and low levels of literacy, yet optimal screening tools suited for these settings are limited (Ferri et al., 2005). Additionally, the HIV/AIDS epidemic will likely significantly alter the patterns of cognition and dementia in late adulthood (Mateen and Mills, 2012). The adapted OCSPlus measure bypasses limitations of paper-and-pencil assessments and builds on the rising familiarity with electronic devices now common in many LMICs. Our study highlights important advantages offered by the OCSPlus instrument in standardization of administration, use of visually oriented and simple language tasks that reduce cultural and literacy limitations and automatic scoring with reduced assessor input or bias, allowing for important gains in speed and process of data collection and management. Overall, this suggests that OCSPlus is suitable as a cognitive screen in a variety of cultural and socioeconomic settings. We believe that our initial findings in this study provide strong argument that OCSPlus hold promise for research purposes and builds a strong foundation for assessing its clinical utility in Kenya. In the context of neurocognitive screening, the medical and financial consequences must be weighed for both screening and full neuropsychological assessments. Given that HAND is quite prevalent, carrying out a gold standard neuropsychological evaluation on every PLWH would be ideal. Nonetheless, this will be prohibitive, and impractical in many clinical settings in SSA. Overburdened and under-resourced health facilities in many cases do not have the time, staff and financial resources to conduct full evaluations on all their clients. Additionally, many HIV clinics in Kenya and similar settings do not have neuropsychologists. Therefore, a screening measure that is brief, easy to use that can assist clinics determine which clients are most likely to have cognitive deficits can help clinics make better referrals, better tracking, integration with electronic medical records and use their limited resources more wisely. While our findings indicate that OCSPlus holds promise as an easy-to-use solution for screening cognition in this population, however, more research is needed to replicate our findings with a larger sample before tool can be widely used in our context. Assessment of its clinical utility is also required before use in the clinical setting. Ideally, such research should be able to clarify which cadre of healthcare providers will provide such services and assess the health systems preparedness, e.g., a robust pathway to care for those identified to have cognitive deficits.

### Limitations and future research

Our study had a few limitations. This being a cross-sectional study, it precludes any conclusions on causality for the associations observed herein. We recruited our OALWH from public HIV clinics, thus, our findings may not be readily generalized to OALWH who may be out of care or attending private or urban HIV clinics or recruited from the community. Additionally, given the relatively small sample size, and modest convergent validity, we recommend further research with larger sample sizes to confirm the tool’s reliability and applicability across different populations and settings. Additional studies are required to establish local normative data, carry out sensitivity and specificity analyses and conduct long-term follow-ups to refine the clinical and community applicability of the tool. The OCSPlus tool, in its current state, is designed to briefly measure more detailed cognitive performance metrics that can be used to make informed clinical decisions and not to separate the spectrum of cognitive deficits into impairment categories. Normative performance data have not been established for OCSPlus in Kenya, making generalization of performance on it difficult. Also, the conventional tests used in our study (the Ravens standard progressive matrices and the international HIV dementia scale) assessed limited cognitive domains compared to the OCSPlus, and this may explain some of the low correlation coefficients for the convergent validity. Future validation studies should expand the conventional neuropsychological battery. Despite these limitations, we believe OCSPlus offers researchers and clinicians a brief, easy-to-use solution to screen for cognitive function among older adults living with HIV and other patients with brain-involving pathologies. However, more research is required to validate the tool as a screening tool for neurocognitive impairments. A larger sample, statistically powered to confirm internal and external validity, is essential for the tool scale-up. There is also scope for in-depth qualitative research with potential users of OCSPlus in clinical settings, e.g., clinicians and nurses, to assess the utility of the tool in these settings.

## Conclusion

Neurocognitive impairment is one of the most common complications of HIV infection, with serious medical and functional impacts. Valid and reliable assessments of HIV-related cognitive deficits are essential to advance research, yet challenging to implement in many settings where research is most urgently needed given that most existing tests were designed to detect only the severe forms of impairments, have poor psychometric properties, require additional equipment (e.g., stopwatches, pens test forms) and many require highly trained professionals to administer, score and interpret – resources not readily available in SSA. Mobile technologies offer better solutions to neurocognitive screening by making testing more accurate, efficient, affordable, and accessible to those who need testing, especially in low-resource settings. Our study provides preliminary evidence for the reliability and validity of OCSPlus, an innovative, domain-specific, and domain-general tablet-based approach to cognition assessment, among a clinic sample of OALWH and their uninfected peers in a low-literacy Kenyan setting. The results show that OCSPlus has promise as a brief cognitive screen in this population, showing good test–retest reliability and convergent validity. Our study also provides an initial understanding of the cognitive performance of older Kenyans by HIV status and the factors associated with the cognitive performance of these adults on the Kenyan coast. Further validation and epidemiologic studies are needed to understand better the utility of the tool and the cognitive function of older adults in the region.

## Data Availability

The raw data supporting the conclusions of this article will be made available by the authors, without undue reservation.

## References

[ref1] Abubakar A. Van De Vijver F. J. (2017). “How to adapt tests for sub-Saharan Africa” in Handbook of applied developmental science in Sub-Saharan Africa. eds. Abubakar A. Vijver F. J. (Cham: Springer), 197–212.

[ref2] Alford K. Vera J. (2018). Cognitive impairment in people living with HIV in the ART era: a review. Br. Med. Bull. 127, 55–68. doi: 10.1093/bmb/ldy019, PMID: 29868901

[ref19] Antinori A. Arendt G. Grant I. Letendre S. Munoz-Moreno J. Eggers C. . (2013). Assessment, diagnosis, and treatment of HIV-associated neurocognitive disorder: a consensus report of the mind exchange program. Clin. Infect. Dis. 56, 1004–1017. doi: 10.1093/cid/cis975, PMID: 23175555 PMC3657494

[ref3] Asiimwe S. B. Farrell M. Kobayashi L. C. Manne-Goehler J. Kahn K. Tollman S. M. . (2020). Cognitive differences associated with HIV serostatus and antiretroviral therapy use in a population-based sample of older adults in South Africa. Sci. Rep. 10, 1–14. doi: 10.1038/s41598-020-73689-7, PMID: 33024208 PMC7539005

[ref4] Asiimwe S. B. Kanyesigye M. Bwana B. Okello S. Muyindike W. (2015). Predictors of dropout from care among HIV-infected patients initiating antiretroviral therapy at a public sector HIV treatment clinic in sub-Saharan Africa. BMC Infect. Dis. 16, 1–10. doi: 10.1186/s12879-016-1392-7, PMID: 26832737 PMC4736127

[ref5] Aung H. L. Aghvinian M. Gouse H. Robbins R. N. Brew B. J. Mao L. . (2021). Is there any evidence of premature, accentuated and accelerated aging effects on neurocognition in people living with HIV? A systematic review. AIDS Behav. 25, 917–960. doi: 10.1007/s10461-020-03053-3, PMID: 33025390 PMC7886778

[ref6] Benjamini Y. Hochberg Y. (1995). Controlling the false discovery rate: a practical and powerful approach to multiple testing. J. Royal Stat. Soc. Methodological 57, 289–300. doi: 10.1111/j.2517-6161.1995.tb02031.x

[ref7] Bernard C. Font H. Diallo Z. Ahonon R. Tine J. M. Abouo F. N.' G. . (2023). Factors associated with verbal fluency in older adults living with HIV in West Africa: A longitudinal study. Trop. Med. Int. Health 28, 35–42. doi: 10.1111/tmi.13830, PMID: 36398852 PMC9812871

[ref8] Bernard C. Font H. Diallo Z. Ahonon R. Tine J. M. Abouo F. N. . (2021). Effects of age, level of education and HIV status on cognitive performance in west African older adults: the West Africa IeDEA cohort collaboration. AIDS Behav. 25, 3316–3326. doi: 10.1007/s10461-021-03309-6, PMID: 34050826 PMC8422594

[ref9] Bloch M. Kamminga J. Jayewardene A. Bailey M. Carberry A. Vincent T. . (2016). A screening strategy for HIV-associated neurocognitive disorders that accurately identifies patients requiring neurological review. Clin. Infect. Dis. 63, 687–693. doi: 10.1093/cid/ciw399, PMID: 27325690 PMC4981762

[ref10] Clifford D. B. Ances B. M. (2013). HIV-associated neurocognitive disorder. Lancet Infect. Dis. 13, 976–986. doi: 10.1016/S1473-3099(13)70269-X, PMID: 24156898 PMC4108270

[ref11] Cysique L. A. Bain M. P. Lane T. A. Brew B. J. (2012). Management issues in HIV-associated neurocognitive disorders. Neurobehav HIV Med 4, 63–73. doi: 10.2147/NBHIV.S30466, PMID: 41133991

[ref12] Demeyere N. Haupt M. Webb S. S. Strobel L. Milosevich E. T. Moore M. J. . (2021). Introducing the tablet-based Oxford Cognitive Screen-Plus (OCS-Plus) as an assessment tool for subtle cognitive impairments. Sci. Rep. 11:8000. doi: 10.1038/s41598-021-87287-8, PMID: 33846501 PMC8041764

[ref13] Ettenhofer M. L. Hinkin C. H. Castellon S. A. Durvasula R. Ullman J. Lam M. . (2009). Aging, neurocognition, and medication adherence in HIV infection. Am. J. Geriatr. Psychiatry 17, 281–290. doi: 10.1097/JGP.0b013e31819431bd, PMID: 19307857 PMC2679810

[ref14] Ferri C. P. Prince M. Brayne C. Brodaty H. Fratiglioni L. Ganguli M. . (2005). Global prevalence of dementia: a Delphi consensus study. Lancet 366, 2112–2117. doi: 10.1016/S0140-6736(05)67889-0, PMID: 16360788 PMC2850264

[ref15] Folstein M. F. Folstein S. E. McHugh P. R. (1975). “Mini-mental state”: a practical method for grading the cognitive state of patients for the clinician. J. Psychiatr. Res. 12, 189–198. doi: 10.1016/0022-3956(75)90026-6, PMID: 1202204

[ref16] Glickman M. E. Rao S. R. Schultz M. R. (2014). False discovery rate control is a recommended alternative to Bonferroni-type adjustments in health studies. J. Clin. Epidemiol. 67, 850–857. doi: 10.1016/j.jclinepi.2014.03.012, PMID: 24831050

[ref17] Gorman A. A. Foley J. M. Ettenhofer M. L. Hinkin C. H. van Gorp W. G. (2009). Functional consequences of HIV-associated neuropsychological impairment. Neuropsychol. Rev. 19, 186–203. doi: 10.1007/s11065-009-9095-0, PMID: 19472057 PMC2871666

[ref18] Gouse H. Masson C. J. Henry M. Marcotte T. D. London L. Kew G. . (2021). Assessing HIV provider knowledge, screening practices, and training needs for HIV-associated neurocognitive disorders. A short report. AIDS Care 33, 468–472. doi: 10.1080/09540121.2020.1736256, PMID: 32138523 PMC7483165

[ref20] Guha A. Brier M. R. Ortega M. Westerhaus E. Nelson B. Ances B. M. (2016). Topographies of cortical and subcortical volume loss in HIV and aging in the cART era. J. Acquir. Immune Defic. Syndr. (1999), 73:374. 27454251 10.1097/QAI.0000000000001111 PMC5085858

[ref21] Haddow L. J. Floyd S. Copas A. Gilson R. J. C. (2013). A systematic review of the screening accuracy of the HIV Dementia Scale and International HIV Dementia Scale. PLoS One 8:e61826. doi: 10.1371/journal.pone.0061826, PMID: 23613945 PMC3628906

[ref22] Harris P. A. Taylor R. Thielke R. Payne J. Gonzalez N. Conde J. G. (2009). Research electronic data capture (REDCap)—a metadata-driven methodology and workflow process for providing translational research informatics support. J. Biomed. Inform. 42, 377–381. doi: 10.1016/j.jbi.2008.08.010, PMID: 18929686 PMC2700030

[ref23] Hays R. D. DiMatteo M. R. (1987). A short-form measure of loneliness. J. Pers. Assess. 51, 69–81. doi: 10.1207/s15327752jpa5101_6, PMID: 3572711

[ref24] Heaton R. K. Franklin D. R. Ellis R. J. McCutchan J. Letendre S. L. Leblanc S. . (2011). HIV-associated neurocognitive disorders before and during the era of combination antiretroviral therapy: differences in rates, nature, and predictors. J. Neurovirol. 17, 3–16. doi: 10.1007/s13365-010-0006-1, PMID: 21174240 PMC3032197

[ref25] Hoops S. Nazem S. Siderowf A. D. Duda J. E. Xie S. X. Stern M. B. . (2009). Validity of the MoCA and MMSE in the detection of MCI and dementia in Parkinson disease. Neurology 73, 1738–1745. doi: 10.1212/WNL.0b013e3181c34b47, PMID: 19933974 PMC2788810

[ref26] Hübner C. M. Demeyere N. Preul C. Finke K. (2020). Validation of the Oxford Cognitive Screen-Plus: Tablet-based cognitive testing in patients with amnestic mild cognitive impairment: Neuropsychology/computerized neuropsychological assessment. Alzheimers Dement. 16:e042557. doi: 10.1002/alz.042557

[ref27] Humphreys G. W. Duta M. D. Montana L. Demeyere N. McCrory C. Rohr J. . (2017). Cognitive function in low-income and low-literacy settings: validation of the tablet-based Oxford cognitive screen in the health and aging in Africa: a longitudinal study of an INDEPTH community in South Africa (HAALSI). J. Gerontology Series B 72, 38–50. doi: 10.1093/geronb/gbw139, PMID: 27974474 PMC5156498

[ref28] Irollo E. Luchetta J. Ho C. Nash B. Meucci O. (2021). Mechanisms of neuronal dysfunction in HIV-associated neurocognitive disorders. Cell. Mol. Life Sci. 78, 4283–4303. doi: 10.1007/s00018-021-03785-y, PMID: 33585975 PMC8164580

[ref29] Iudicello J. E. Woods S. P. Cattie J. E. Doyle K. Grant I. HIV Neurobehavioral Research Program Group (2013). Risky decision-making in HIV-associated neurocognitive disorders (HAND). Clin. Neuropsychol. 27, 256–275. doi: 10.1080/13854046.2012.740077, PMID: 23181946 PMC3609907

[ref30] Jenkins D. G. Quintana-Ascencio P. F. (2020). A solution to minimum sample size for regressions. PLoS One 15:e0229345. doi: 10.1371/journal.pone.0229345, PMID: 32084211 PMC7034864

[ref31] Katz M. H. (2011). Multivariable analysis: a practical guide for clinicians and public health researchers. Cambridge: Cambridge University Press.

[ref32] Kenya National Bureau of Statistics (2019). 2019 Kenya Population and Housing Census Volume III: Distribution of Population by Age and Sex. Nairobi: Kenya National Bureau of Statistics.

[ref33] Kenya National Bureau of Statistics. (2019). The 2019 Kenya Population and Housing Census. Volume I: Population by county and sub-county. Available online at:http://housingfinanceafrica.org/app/uploads/VOLUME-I-KPHC-2019.pdf (accessed March 9, 2022).

[ref34] Kobayashi L. C. Mateen F. J. Montana L. Wagner R. G. Kahn K. Tollman S. M. . (2019). Cognitive function and impairment in older, rural south African adults: evidence from “health and aging in Africa: a longitudinal study of an INDEPTH Community in Rural South Africa”. Neuroepidemiology 52, 32–40. doi: 10.1159/000493483, PMID: 30476911 PMC6531856

[ref35] Lazar M. Moroti R. Barbu E. C. Chitu-Tisu C. E. Tiliscan C. Erculescu T. M. . (2024). The Impact of HIV on Early Brain Aging—A Pathophysiological (Re) View. J. Clin. Med. 13:7031. doi: 10.3390/jcm13237031, PMID: 39685490 PMC11642420

[ref36] Livingston G. Huntley J. Sommerlad A. Ames D. Ballard C. Banerjee S. . (2020). Dementia prevention, intervention, and care: 2020 report of the Lancet Commission. Lancet 396, 413–446. doi: 10.1016/S0140-6736(20)30367-6, PMID: 32738937 PMC7392084

[ref37] Mateen F. J. Mills E. J. (2012). Aging and HIV-related cognitive loss. JAMA 308, 349–350. doi: 10.1001/jama.2012.8538, PMID: 22820786

[ref38] Mekuriaw B. Belayneh Z. Teshome W. Akalu Y. (2023). Prevalence and variability of HIV/AIDS-associated neurocognitive impairments in Africa: a systematic review and meta-analysis. BMC Public Health 23:997. doi: 10.1186/s12889-023-15935-x, PMID: 37254121 PMC10228136

[ref39] Michael H. U. Naidoo S. Mensah K. B. Ramlall S. Oosthuizen F. (2021). The impact of antiretroviral therapy on neurocognitive outcomes among people living with HIV in low-and middle-income countries (LMICs): a systematic review. AIDS Behav. 25, 492–523. doi: 10.1007/s10461-020-03008-8, PMID: 32851562

[ref40] Mwangala P. N. Mabrouk A. Wagner R. Newton C. R. J. C. Abubakar A. A. (2021). Mental health and well-being of older adults living with HIV in sub-Saharan Africa: a systematic review. BMJ Open 11:e052810. doi: 10.1136/bmjopen-2021-052810 PMC8461287 34551953

[ref41] Mwangala P. N. Nasambu C. Wagner R. G. Newton C. R. Abubakar A. (2022). Prevalence and factors associated with mild depressive and anxiety symptoms in older adults living with HIV from the Kenyan coast. J. Int. AIDS Soc. 25:e25977. doi: 10.1002/jia2.25977, PMID: 36176027 PMC9522642

[ref45] Mwangala P. N. Nasambu C. Wagner R. G. Newton C. R. Abubakar A. (2024). Prevalence and factors associated with frailty among older adults living with HIV compared to their uninfected peers from the Kenyan Coast. Int. J. Public Health 69:1606284. doi: 10.3389/ijph.2024.1606284 38426187 PMC10901986

[ref42] Mwangala P. N. Newton C. R. Abas M. Abubakar A. (2018). Screening tools for HIV-associated neurocognitive disorders among adults living with HIV in sub-Saharan Africa: a scoping review. AAS Open Res. 1:1. doi: 10.12688/aasopenres.12921.1, PMID: 31844836 PMC6914359

[ref43] Mwangala P. N. Wagner R. G. Newton C. R. Abubakar A. (2023a). Strategies for improving mental health and wellbeing used by adults ageing with HIV from the Kenyan coast: a qualitative exploration. Wellcome Open Res. 7:221. doi: 10.12688/wellcomeopenres.18212.2, PMID: 37415804 PMC10320323

[ref44] Mwangala P. N. Wagner R. G. Newton C. R. Abubakar A. (2023b). Navigating life with HIV as an older adult on the Kenyan coast: perceived health challenges seen through the biopsychosocial model. Int. J. Public Health 68:1605916. doi: 10.3389/ijph.2023.1605916, PMID: 37398632 PMC10308997

[ref46] Nasreddine Z. S. Phillips N. A. Bédirian V. Charbonneau S. Whitehead V. Collin I. . (2005). The Montreal Cognitive Assessment, MoCA: a brief screening tool for mild cognitive impairment. J. Am. Geriatr. Soc. 53, 695–699. doi: 10.1111/j.1532-5415.2005.53221.x, PMID: 15817019

[ref47] National AIDS Control Council (NACC) (2016). Kenya HIV County Profiles. New Delhi: National AIDS Control Council (NACC).

[ref48] Nyongesa M. K. Mwangala P. N. Mwangi P. Kombe M. Newton C. R. J. C. Abubakar A. A. (2018). Neurocognitive and mental health outcomes and association with quality of life among adults living with HIV: a cross-sectional focus on a low-literacy population from coastal Kenya. BMJ Open 8:e023914. doi: 10.1136/bmjopen-2018-023914, PMID: 30224402 PMC6144406

[ref49] Palmore E. (2001). The ageism survey: First findings. The Gerontologist 41, 572–575. doi: 10.1093/geront/41.5.572 11574698

[ref50] Raven J. C. Court J. H. Raven J. (1977). Manual for Raven's progressive matrices and vocabulary scales. The coloured progressive matrices. London: Raven, J.C.Lewis, H.K.

[ref51] Reinius M. Wettergren L. Wiklander M. Svedhem V. Ekström A. M. Eriksson L. E. (2017). Development of a 12-item short version of the HIV stigma scale. Health Qual. Life Outcomes 15, 1–9. doi: 10.1186/s12955-017-0691-z, PMID: 28558805 PMC5450123

[ref52] Roberts R. Vohora R. Webb S. S. Demeyere N. (2024). Validating the OCS-Plus against a clinical standard: A brief report. J. Neuropsychol. 18, 452–458. doi: 10.1111/jnp.12369 38654444

[ref53] Rotenberg S. Ruthralingam M. Hnatiw B. Neufeld K. Yuzwa K. E. Arbel I. . (2020). Measurement properties of the multiple errands test: A systematic review. Arch. Phys. Med. Rehabil. 101, 1628–1642. doi: 10.1016/j.apmr.2020.01.019 32113973

[ref54] Rowe K. Duta M. Demeyere N. Wagner R. G. Pettifor A. Kahn K. . (2021). Validation of Oxford Cognitive Screen: Executive Function (OCS-EF), a tablet-based executive function assessment tool amongst adolescent females in rural South Africa. Int. J. Psychol. 56, 895–907. doi: 10.1002/ijop.12764, PMID: 33951197

[ref55] Royston P. Moons K. G. M. Altman D. G. Vergouwe Y. (2009). Prognosis and prognostic research: developing a prognostic model. BMJ 338:b604. doi: 10.1136/bmj.b604, PMID: 19336487

[ref56] Sacktor N. C. Wong M. Nakasujja N. Skolasky R. L. Selnes O. A. Musisi S. . (2005). The International HIV Dementia Scale: a new rapid screening test for HIV dementia. AIDS 19, 1367–1374. doi: 10.1097/01.aids.0000180790.77379.3a, PMID: 16103767

[ref57] Scutari R. Alteri C. Perno C. Svicher V. Aquaro S. (2017). The role of HIV infection in neurologic injury. Brain Sci. 7:38. doi: 10.3390/brainsci7040038, PMID: 28383502 PMC5406695

[ref58] Shah A. Gangwani M. R. Chaudhari N. S. Glazyrin A. Bhat H. K. Kumar A. (2016). Neurotoxicity in the post-HAART era: caution for the antiretroviral therapeutics. Neurotox. Res. 30, 677–697. doi: 10.1007/s12640-016-9646-0, PMID: 27364698 PMC5050092

[ref59] Sheppard D. P. Woods S. P. Bondi M. W. Gilbert P. E. Massman P. J. Doyle K. L. . (2015). Does older age confer an increased risk of incident neurocognitive disorders among persons living with HIV disease? Clin. Neuropsychol. 29, 656–677. doi: 10.1080/13854046.2015.1077995, PMID: 26367342 PMC4570033

[ref60] Thames A. D. Streiff V. Patel S. M. Panos S. E. Castellon S. A. Hinkin C. H. (2012). The role of HIV infection, cognition, and depression in risky decision-making. J. Neuropsychiatry Clin. Neurosci. 24, 340–348. doi: 10.1176/appi.neuropsych.11110340, PMID: 23037648 PMC3608191

[ref61] Tozzi V. Balestra P. Galgani S. Murri R. Bellagamba R. Narciso P. . (2003). Neurocognitive performance and quality of life in patients with HIV infection. AIDS Res. Hum. Retrovir. 19, 643–652. doi: 10.1089/088922203322280856, PMID: 13678465

[ref62] UNAIDS. (2022). Global HIV & AIDS Statistics — 2022 Fact Sheet. Available online at: https://www.unaids.org/sites/default/files/media_asset/UNAIDS_FactSheet_en.pdf (accessed June 13, 2023).

[ref63] Üstün T. B. Chatterji S. Kostanjsek N. Rehm J. Kennedy C. Epping-Jordan J. . (2010). Developing the World Health Organization disability assessment schedule 2.0. Bull. World Health Organ. 88, 815–823. doi: 10.2471/BLT.09.067231, PMID: 21076562 PMC2971503

[ref64] Vivithanaporn P. Heo G. Gamble J. Krentz H. B. Hoke A. Gill M. J. . (2010). Neurologic disease burden in treated HIV/AIDS predicts survival: a population-based study. Neurology 75, 1150–1158. doi: 10.1212/WNL.0b013e3181f4d5bb, PMID: 20739646 PMC3013488

[ref65] Webb S. S. Hobden G. Roberts R. Chiu E. G. King S. Demeyere N. (2022). Validation of the UK English Oxford cognitive screen-plus in sub-acute and chronic stroke survivors. Eur. Stroke J. 7, 476–486. doi: 10.1177/23969873221119940, PMID: 36478766 PMC9720845

[ref66] Yurdugül H. (2008). Minimum sample size for Cronbach’s coefficient alpha: A Monte-Carlo study. Hacettepe Üniversitesi eğitim fakültesi dergisi 35, 1–9.

[ref67] Zenebe Y. Necho M. Yimam W. Akele B. (2022). Worldwide occurrence of HIV-associated neurocognitive disorders and its associated factors: A systematic review and meta-analysis. Front. Psychiatry 13:814362. doi: 10.3389/fpsyt.2022.814362 35711575 PMC9193596

